# Interplay of niche and respiratory network in shaping bacterial colonization

**DOI:** 10.1016/j.jbc.2024.108052

**Published:** 2024-12-09

**Authors:** Stuti Srivastav, Arpita Biswas, Amitesh Anand

**Affiliations:** Department of Biological Sciences, Tata Institute of Fundamental Research, Mumbai, Maharashtra, India

**Keywords:** bacterial pathogenesis, respiratory chain, metabolic plasticity, bacterial metabolism, bioenergetics

## Abstract

The human body is an intricate ensemble of prokaryotic and eukaryotic cells, and this coexistence relies on the interplay of many biotic and abiotic factors. The inhabiting microbial population has to maintain its physiological homeostasis under highly dynamic and often hostile host environments. While bacterial colonization primarily relies on the metabolic suitability for the niche, there are reports of active remodeling of niche microenvironments to create favorable habitats, especially in the context of pathogenic settlement. Such physiological plasticity requires a robust metabolic system, often dependent on an adaptable energy metabolism. This review focuses on the respiratory electron transport system and its adaptive consequences within the host environment. We provide an overview of respiratory chain plasticity, which allows pathogenic bacteria to niche-specify, niche-diversify, mitigate inflammatory stress, and outcompete the resident microbiota. We have reviewed existing and emerging knowledge about the role of respiratory chain components responsible for the entry and exit of electrons in influencing the pathogenic outcomes.

The human body presents a highly complex ecosystem for colonizing microbes. The upper airway tissues have near atmospheric oxygen levels, while the gastrointestinal lumen lacks oxygen (1). A similar extreme is observed in the pH variations across tissues, with a pH of ≈2 in the stomach to above seven in the liver, heart, and several other organs (2, 3). The situation is further complicated due to the changes in the microenvironment of the same tissue type (2). Therefore, a significant spatiotemporal variation exists in the composition of the human microbiome (4).

While most commensal bacteria are adapted to thrive within particular tissue environments, many pathogens can successfully colonize diverse organs (5, 6, 7). When a pathogen encounters a human host, it confronts various challenges before establishing a successful infection (Fig. 1*A*) (8). These include temperature fluctuations, pH changes, host immune responses, and competition from resident microbiota (9). Pathogenic success in hostile environments requires meeting metabolic demands by maintaining energy homeostasis (Fig. 1*B*) (9). Notably, several *in vitro* studies have reported the maintenance of energy reserve in the form of intracellular adenylate charge across varying ambient conditions (10, 11, 12). The degeneracy in bacterial respiro-fermentative energetics allows conditional flux re-routing to adopt an optimal metabolic state conducive to the microenvironment (13, 14, 15). Owing to the associated complexity, the understanding of host-resident bacterial bioenergetics remained primarily phenomenological and associative. However, several recent studies have provided mechanistic insights.

**Figure 1:**
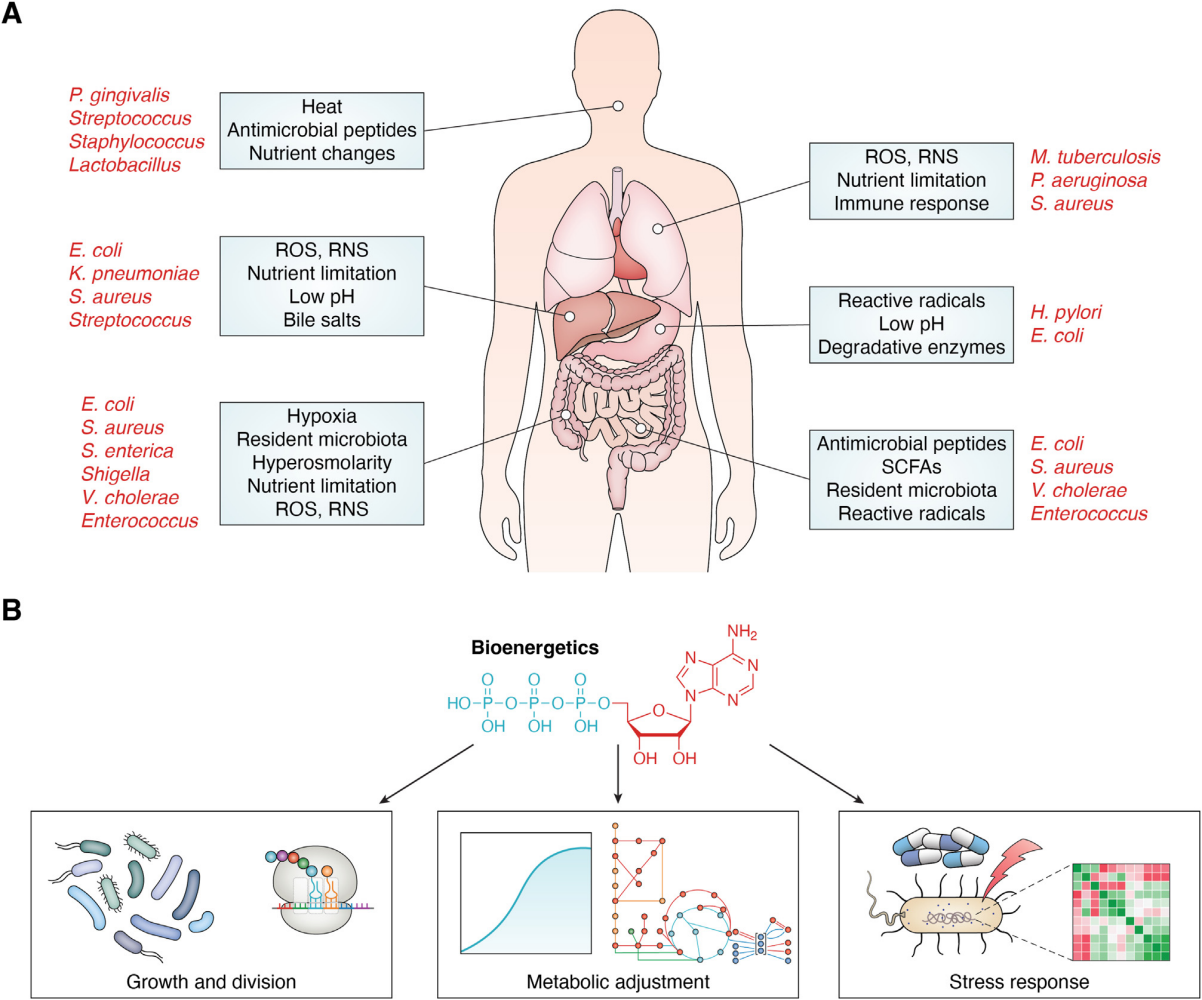
**The interplay between niche colonization and bioenergetics**. *A*, the diverse stresses a pathogen may encounter while invading a specific organ or tissue niche are shown in blue boxes. Some pathogenic bacteria capable of successfully invading these niches are also depicted (9, 118, 119, 120, 121). *B*, bioenergetics plays a critical role in regulating processes central to bacterial pathophysiology. It supports cellular processes required for niche adaptation like growth and division, metabolic adjustment, and stress response (122, 123, 124). ROS, reactive oxygen species; RNS, reactive nitrogen species; SCFAs, short chain fatty acids.

*Porphyromonas gingivalis* pathogenesis is an illustrative example of the interplay between niche dynamics and respiratory modulation in shaping pathogenic outcomes. *P. gingivalis*, an anaerobe, is found below the gum line in humans and is implicated in causing physiological changes that enable the onset of periodontitis (16). *P. gingivalis* has an asaccharolytic, proteolytic lifestyle in the buccal niche, utilizing the amino acid components of human serum as a carbon source (17, 18). Under this metabolic state, *P. gingivalis* lowers the expression of fimbrial genes involved in surface adhesion and biofilm formation. However, a build-up of pyruvate alongside lactate due to physiological conditions such as inflammation, stress, diet, infection, *and so on*, causes transition to a biofilm state (Fig. 2*A*) (17, 19, 20, 21). Here, pyruvate is used to support gluconeogenesis, which provides precursors for the Pentose Phosphate Pathway (PPP). In turn, PPP provides precursors for anabolic reactions. Parallely, lactate fermentation helps maintain the redox balance of cells through NADH oxidation. However, the underlying resource demand and allocation dictating the flux through these pathways remain poorly studied. The metabolic coupling of lactate and pyruvate catabolism triggers the expression of fimbrial adhesion genes in *P. gingivalis*. This metabolic switch leads to surface adhesion and, eventually, biofilm formation, which precedes the pathology of periodontitis (17). Such niche, nutrient, and network intricacies define pathogenic outcomes. Therefore, a comprehensive understanding of bioenergetic adaptability is critical to delineate the metabolic basis of bacterial pathogenesis.

**Figure 2:**
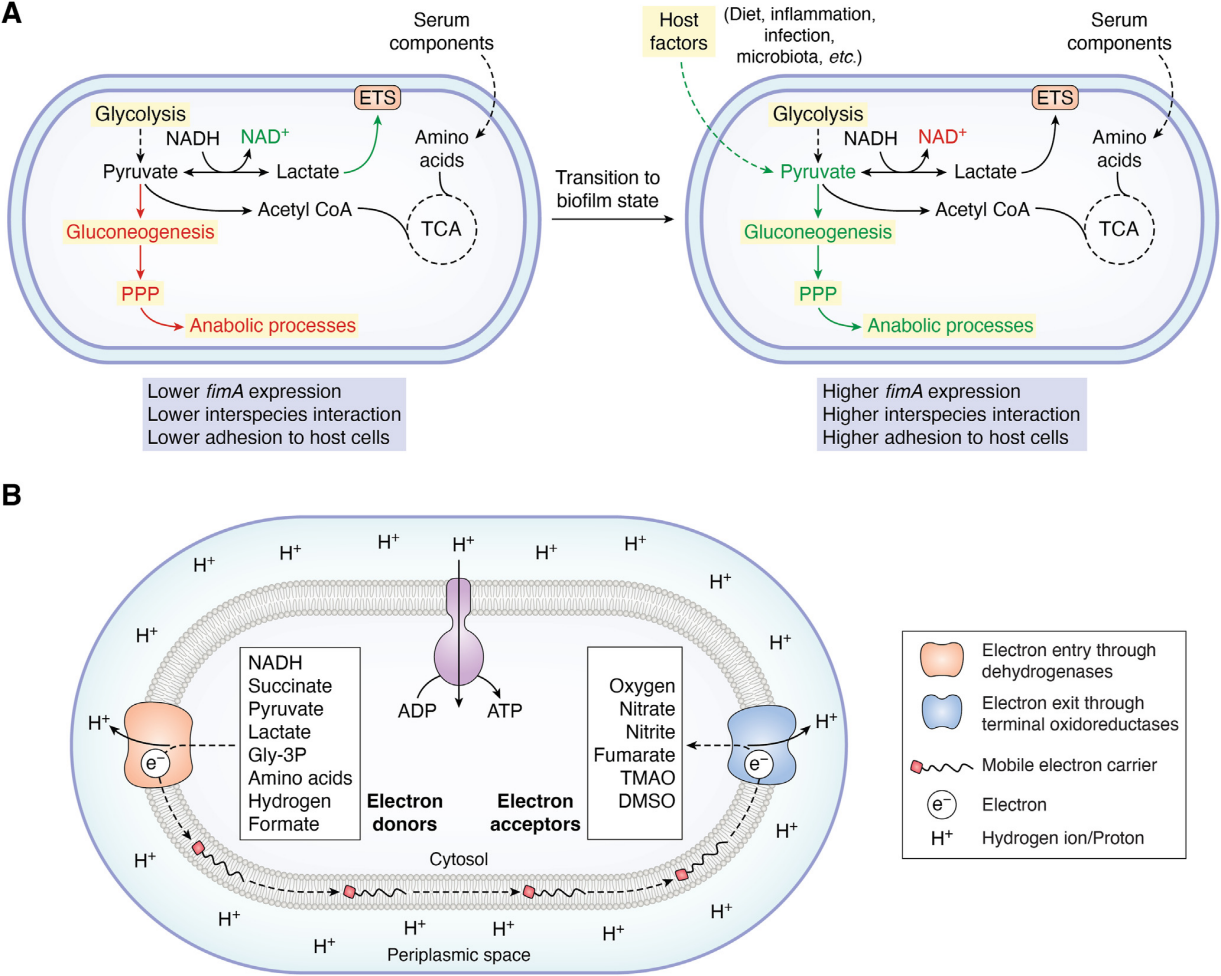
**Metabolic and respiratory chain plasticity is central to pathogenesis**. *A*, *Porphyromonas gingivalis* undergoes a metabolic transition, forming a biofilm due to exogenous pyruvate availability. Upregulated pathways and metabolites are depicted in *green*, downregulated pathways and metabolites are depicted in *red*, and unaltered pathways and metabolites are shown in *black*. The presence of exogenous pyruvate shifts central carbon metabolism towards gluconeogenesis and anabolism. This causes a change in gene expression, particularly leading to higher expression of fimbrial adhesion genes (*fimA*), which is essential for biofilm formation (24). *B*, the respiratory chain of bacteria is highly flexible. Electrons enter the respiratory chain through dehydrogenases that oxidize the cognate electron donor. Various examples of electron donors are depicted in the figure. These electrons are passed through various dehydrogenases to the mobile electron carriers that shuttle them to the terminal oxidoreductases. These oxidases utilize the received electrons to reduce their cognate terminal electron acceptor. Examples of terminal electron acceptors are depicted in the figure. During electron transfer, the reduction potential-favored flow of electrons provides energy to pump protons into the periplasmic space. This creates a transmembrane proton motive force which is used to drive ATP synthesis *via* the ATP synthase (22, 23, 27). Gly-3P, glycerol-3-phosphate; TMAO, trimethylamine N-oxide.

## Respiration and pathogenesis

The respiratory Electron Transport System (ETS) exploits the reduction potential-favored flow of electrons through a chain of enzymes facilitated by mobile carriers to create a Proton Motive Force (PMF) while oxidizing cellular reduced metabolites (Fig. 2*B*) (22, 23, 24). ETS consists of interdependent redox reactions splitting the sizeable free energy of the terminal electron donor-acceptor pair into smaller fragments so that the energy released may be used to pump out protons (25). Throughout evolution, this system has acquired enormous compositional diversity. Therefore, bacterial ETS can receive electrons from several metabolites using a range of dehydrogenases and pass them on to terminal electron acceptors through various oxidoreductases (Fig. 2*B*).

**Figure 3:**
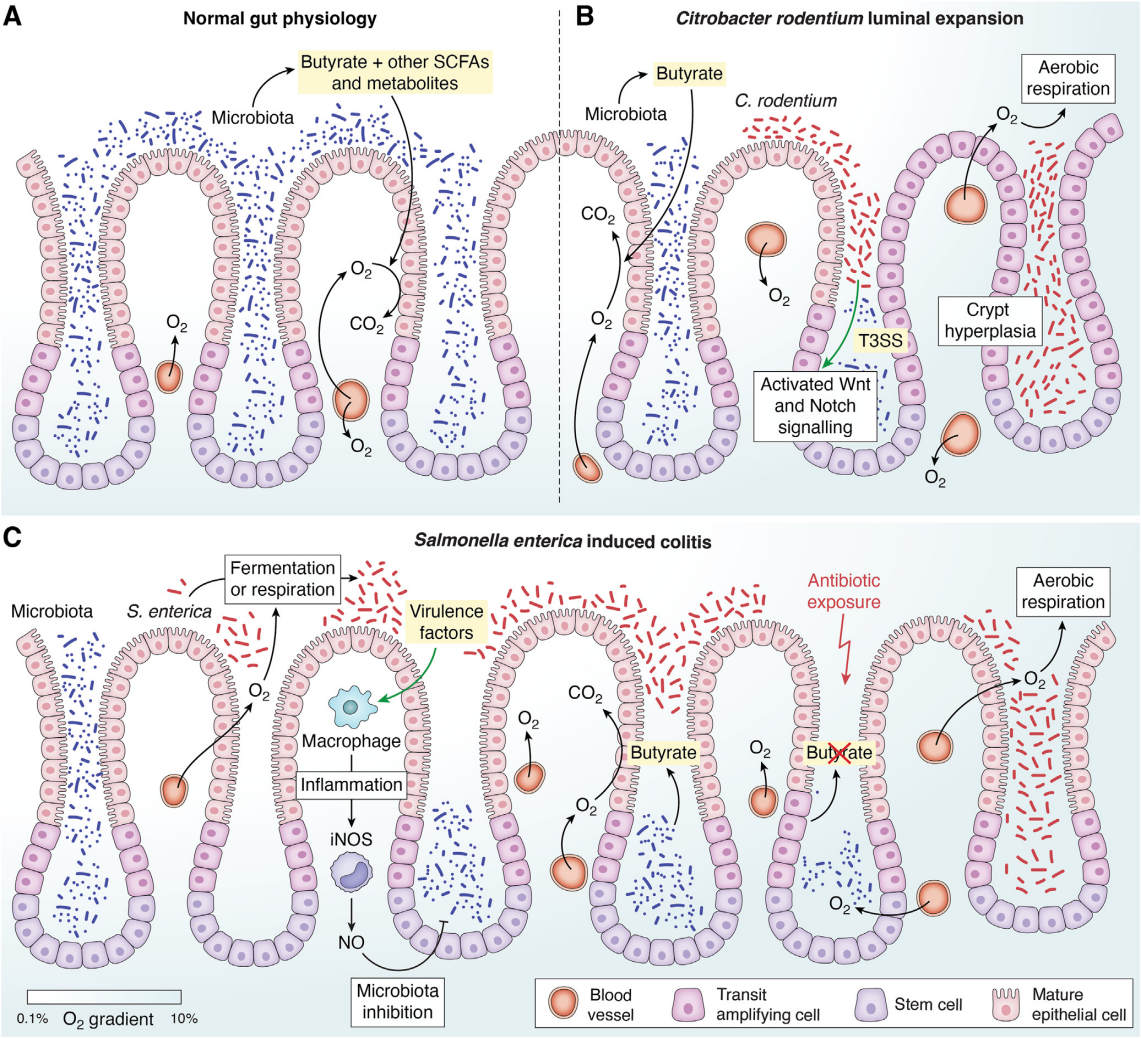
**Efficient respiration is central to bacterial pathophysiology**. *A*, under normal physiological conditions, the intestinal colon consists of mature epithelial cells derived from undifferentiated stem cells in the crypts of the villi. In the lumen of the colon, a dense microbiota utilizes available substrates to produce metabolites like short-chain fatty acids (SCFAs). In particular, butyrate produced by gut-commensal Firmicutes is utilized by mature epithelial cells to perform butyrate fermentation. This fermentation is oxygen-dependent. Since the stem cells do not perform butyrate fermentation, the microenvironment of the crypts is less hypoxic than that of the epithelial surface (32, 33). *B*, *Citrobacter rodentium* triggers an active increase in luminal oxygen concentration through crypt hyperplasia. Upon infection, *C. rodentium* injects effector proteins into the crypt cells through a Type 3 Secretion System (T3SS), activating the Wnt and Notch signaling pathway in these stem cells. This leads to cell proliferation without differentiation, causing a build-up of transit amplifying cells at the surface of the villi, hence resulting in crypt hyperplasia. These cells do not perform butyrate fermentation and consequently, the local oxygen concentration increases. The pathogen uses this oxygen to perform aerobic respiration and proliferate further (34). *C*, during invasion by *Salmonella enterica,* the available oxygen and organic compounds in the lumen are initially used to drive pathogen expansion. As a consequence of infection, the innate immune response of the host is triggered, leading to the expression of inducible nitric oxide synthase (iNOS) in immune cells. The nitric oxide (NO) produced leads to inhibition of not only the pathogen, but also the commensal gut microbes. This reduces the colonization resistance towards *S. enterica*, allowing more pathogen proliferation (35). When an antibiotic is introduced, it can further deplete the commensal bacteria, including the butyrate-producing Firmicutes. Consequently, the lowered butyrate concentration in the gut disables epithelial cells from performing fermentation. This increases the local oxygen concentration. *S. enterica* can utilize this increased oxygen to perform aerobic respiration and colonize the gut successfully (33).

The capabilities of ETS improve multi-fold in the presence of oxygen due to its high reduction potential, which expands the redox potential gap of terminal donor-acceptor significantly (26, 27). However, certain host niches, like the gut, are oxygen deficient. The anaerobic environment of the gut primarily supports obligate anaerobes (28, 29, 30). Several pathogens with facultative anaerobic lifestyles can tailor the environment to establish a competitive bioenergetic advantage (Fig. 3*A*). For instance, colitis caused by *Citrobacter rodentium* drives luminal oxygenation through increased proliferation of intestinal stem cells (31). The colonic resident microbiota produces the metabolic by-product butyrate which is the preferred carbon source for mature colonocytes. These epithelial cells at the surface of intestinal villi consume oxygen to support butyrate fermentation. However, the stem cells in the villi crypts do not perform butyrate fermentation (32, 33). During *C. rodentium* infection, a Type 3 Secretion System (T3SS) injects bacterial effector proteins into villi crypts, leading to increased Wnt and Notch signaling in crypt stem cells. This results in uncontrolled proliferation of these stem cells. Consequently, the undifferentiated transit amplifying cells appear on the villi surface, replacing the mature epithelial cells. The decrease in oxygen-consuming epithelial cells results in local buildup of oxygen. The pathogen can now utilize oxygen to support aerobic respiration through high-oxygen-affinity oxidases and outcompete the resident microbiota (Fig. 3*B*) (34).

Contrary to the active remodeling of the microenvironment, *Salmonella enterica* takes passive advantage of niche oxygenation resulting from the innate immune response and antibiotic exposure. The activated host immune cells release nitric oxide (NO) that can lead to depletion of the resident microbiota. This decreases the microbiota-mediated colonization resistance towards *S. enterica.* At the same time, the fermentation of short-chain fatty acids like butyrate by *S. enterica* limits oxygen-dependent butyrate metabolism by mature colonocytes, causing a decrease in local oxygen consumption. At this stage, *S. enterica* can proliferate using high-oxygen affinity terminal oxidases (Fig. 3*C*) (35). It has also been shown that streptomycin treatment, leading to the loss of butyrate-producing *Clostridium* spp. in the gut, increases oxygenation of the gut epithelium (33). Under these conditions as well, *S. enterica* uses high oxygen affinity terminal oxidase-based respiration, leading to aerobic luminal expansion (Fig. 3*C*). An efficient respiration is, thus, central to successful pathogenesis for various bacteria.

This review examines bacterial respiratory plasticity and degeneracy, emphasizing their pathogenic implications. Bacterial respiratory plasticity implies that at every node of the respiratory chain, a variety of enzymes or mobile carriers can accomplish similar functions. While this plastic arrangement appears to have a local redundancy, there are distinct systems-level influences of various respiratory components (14, 27). The bioenergetic strategies influence cellular resource allocation, especially in the context of the limited resource-carrying capacities of bacteria (36, 37). This partitioning of resources requires tailoring metabolic capabilities to support growth or stress tolerance, popularly known as the fear-greed tradeoff (38, 39, 40). Alteration in respiratory pathways perturbs ATP production, formation of reactive radicals, intracellular redox balance, cellular motility, transport, and transmembrane ion potential, processes central to the bacterial pathophysiology (41, 42, 43, 44, 45). The systemic operation of this system through a robust respiratory chain is critical to establish successful infection. The following sections detail the two terminal ETS components involved in the entry and exit of respiratory electrons in the context of their adaptive consequences within a host.

### Respiratory dehydrogenases and virulence

The respiratory electron flow begins with the transfer of electrons from various reduced metabolites to cognate dehydrogenases. Bacterial membranes harbor an array of dehydrogenases differing in (1) the electron donor they utilize (2), their proton translocation abilities, and (3) their general structure (27). While substrate availability appears to be the primary selection determinant among different classes of dehydrogenases, the niche and associated proteome demand also influence the preference of structurally different dehydrogenases (27, 46). This systems-level degeneracy makes respiratory dehydrogenases a critical factor influencing bacterial pathogenesis.

NADH is the primary reduced metabolite generated through central carbon metabolism, which transfers electrons to the ETS (47). These electrons enter the respiratory chain through NADH dehydrogenases. There are two major types of NADH dehydrogenases based on their ability to pump protons to the periplasmic space. Type I NADH dehydrogenases are large, multisubunit complexes that pump two protons into the periplasmic space for every electron passed over to the lipoquinones. Type II NADH dehydrogenases, on the other hand, are smaller, single-subunit enzymes that do not pump any protons. However, they may provide other advantages, such as a higher reaction turnover and a lower requirement of membrane space (48, 49, 50). This uncoupled electron transfer by type II dehydrogenases is advantageous for many bacteria under aerobiosis in avoiding excess PMF generation (51, 52). Bacteria often contain both types of dehydrogenases, potentially for distinct physiological roles (27).

*Mycobacterium tuberculosis* (Mtb) genome harbors both classes of NADH dehydrogenases (one proton-pumping and two non-proton-pumping dehydrogenases) (53). While these enzymes serve the redundant function of regenerating NAD^+^, they have a distinct impact on mycobacterial pathophysiology. The difference in proton-pumping coupled with NADH oxidation provides distinct advantages on various carbon sources. The two non-proton-pumping NADH dehydrogenases in Mtb, Ndh and NdhA, are essential when grown on long-chain fatty acids since this carbon source demands higher NADH to NAD^+^ turnover (53, 54). When only type I NADH dehydrogenase is functioning, the oxidation of long-chain fatty acids can cause an over-accumulation of protons in the periplasmic space which can be growth inhibitory. This is also evident by the decreased *in vivo* pathogenic potential of Ndh- and NdhA-deficient strain (46). The *in vivo* tolerance to reactive oxygen species (ROS) is also abrogated in Mtb strains lacking Ndh. This is pertinent because a primary macrophage-mediated immune response is the respiratory burst, which produces an abundance of ROS that clears pathogens (55). The presence of Ndh thus becomes necessary for pathogen survival under these conditions. Interestingly, the structurally similar non-proton-pumping NdhA has not been reported to have such an impact (53). While Type I NADH dehydrogenase, Nuo, is not essential for survival outside the host in the presence of Ndh and NdhA, this dehydrogenase is critical for proliferation within macrophages. The deletion mutant of *nuoG*, a gene from the *nuo* operon, has a significantly reduced ability to inhibit macrophage apoptosis and shows lower virulence (56). The complex interplay between these NADH dehydrogenases provides Mtb the required metabolic flexibility for successful infection. Mtb lacking both type I and type II NADH dehydrogenases do not survive *in vivo* (53), likely due to impaired redox homeostasis (57). It is attractive to consider a combination therapy that targets both types of NADH dehydrogenases of the pathogen. While proton-pumping NADH dehydrogenases are widely conserved, targeting type II NADH dehydrogenases can be selective due to their absence from mammalian mitochondria.

The diversity of NADH dehydrogenases is also critical for the pathogenic life cycles of various other bacteria, especially for pathogens causing systemic infections. *Staphylococcus aureus* has two type II NADH dehydrogenases, both of which are required for successful infection in the cardiac, hepatic, and spleen tissues (58). In another opportunistic pathogen, *Streptococcus agalactiae*, a type II- and a sodium-dependent-NADH dehydrogenase (Nqr) are necessary to invade the renal and cardiac tissues; however, only Nqr is involved in colonization of the spleen (51). Yet in another nosocomial pathogen, *Pseudomonas aeruginosa*, the loss of Nqr and type II dehydrogenase causes increased virulence inside macrophages. However, only loss of Nqr is associated with faster disease progression *in vivo* and a higher bacterial burden in the lungs (59). This pathogen-specific bias in exploiting distinct dehydrogenases while colonizing similar habitats highlights the intricate interplay between metabolic networks and the surrounding milieu. With recent research, the importance of plasticity at this node has become increasingly apparent. However, mechanistic insights into these observed phenotypes are lacking. Particularly, the reasons for systemic preference of one type of NADH dehydrogenase over another for distinct disease stages are poorly understood.

The respiratory dehydrogenases utilizing reduced metabolites other than NADH aid in colonizing different tissues or the same tissue with a dynamic microenvironment (27). During early infection, the host's innate immune response in many tissues, like the heart and kidneys, causes activation of inducible Nitric Oxide Synthase (iNOS), producing nitric oxide (NO) (60). In such a microenvironment, NO-resistant metabolic flux can provide an adaptive advantage. *S. aureus* utilizes lactate-quinone oxidoreductase (Lqo) in such inflammatory conditions. Lqo delivers a dual benefit in the cardiac niche as it enables proliferation in a NO-rich environment and exploits the local abundance of lactate to support oxidative phosphorylation (61). Along with Lqo, malate-quinone oxidoreductase (Mqo) is vital for colonization by *S. aureus*. Mqo is an interesting enzyme as it directly links the TCA cycle with ETS and can enable the exploitation of peptide-derived amino acids, another abundant metabolite in these niches. As Lqo enables robust respiration, Mqo can effectively support this process by linking the TCA cycle flux to the respiratory chain. This concerted activity of Lqo and Mqo allows *S. aureus* to avoid reliance on NO-sensitive enzymes (62, 63). This adaptive strategy is an elegant example of exploiting an otherwise hostile environment for pathogenic success. Along the same lines, various studies show that gut-inhabiting pathogens such as *S. enterica* and *Helicobacter pylori* can use molecular hydrogen as an electron donor through hydrogen oxidoreductases, and their infectivity gets compromised without this hydrogenase (64, 65). Notably, the metabolism of gut resident microbiota creates a hydrogen-rich environment (66). Interestingly, the two pathogens spatially segregate within the gut, with *S. enterica* occupying the microbe-dense lower gastrointestinal tract, whereas *H. pylori* tends to infect the microbe-sparse stomach (67, 68).

**Figure 4:**
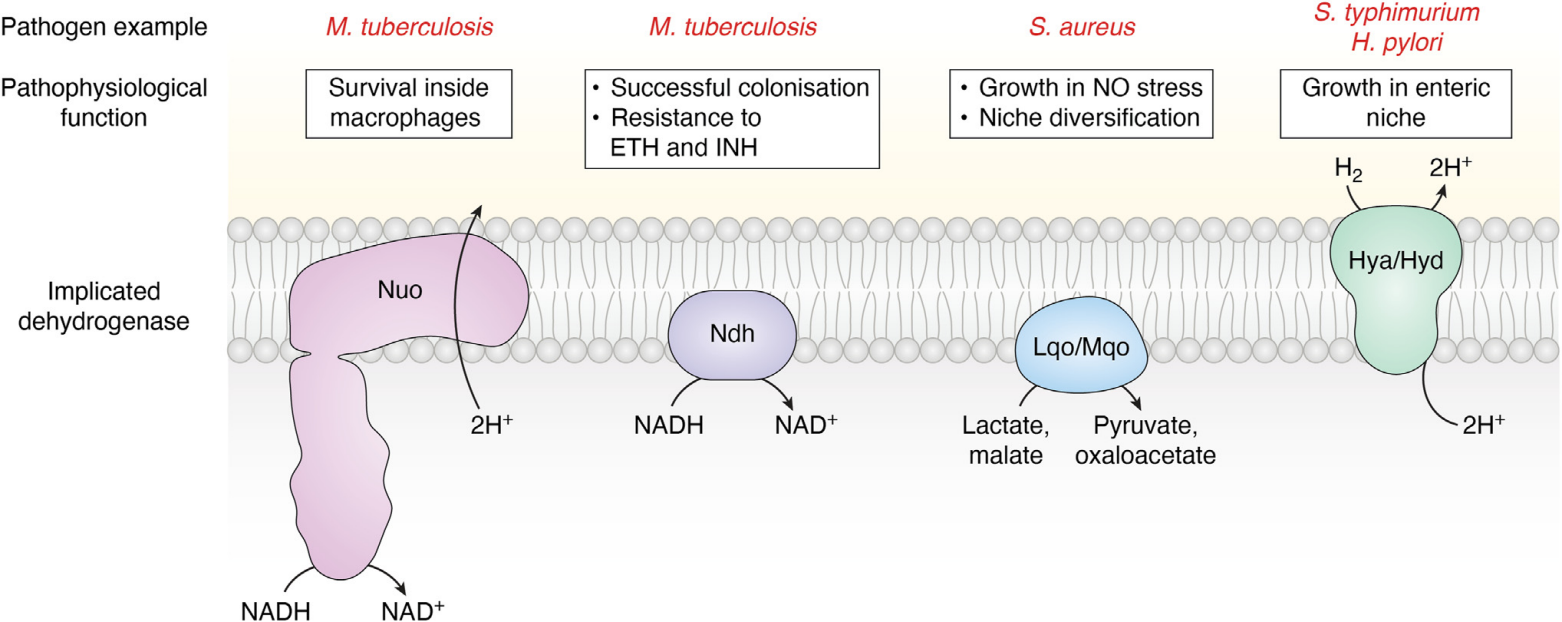
**Plasticity of respiratory dehydrogenases is essential for pathogenesis**. Select examples of types of dehydrogenases are shown as cartoon depictions. The pathogens and the specific roles these enzymes play in these bacteria during the infection cycle are highlighted. Type I NADH dehydrogenase (Nuo) of *Mycobacterium tuberculosis* is essential for survival inside macrophages, while type II NADH dehydrogenase (Ndh) is necessary for host tissue colonization and is associated with resistance development towards anti-TB drugs such as isoniazid (INH) and ethionamide (ETH) (53, 54, 56). In *Staphylococcus aureus*, both lactate-quinone oxidoreductase (Lqo) and malate-quinone oxidoreductase (Mqo) allow optimal utilization of available substrates and promote systemic infection. Pathogens invading the gastrointestinal tract, such as *Salmonella enterica* and *Helicobacter pylori*, exploit the hydrogen present in this niche as an electron donor *via* respiratory hydrogenases (Hya/Hyd) (62, 64, 65).

The flexibility in respiratory dehydrogenase usage allows the capitalization of a plethora of electron donors suited to the niche or metabolic state (Fig. 4). The pathophysiological significance of these dehydrogenases has aptly made them attractive drug targets (69). Several compounds such as phenothiazines are being explored for their potent activity against type II NADH dehydrogenases (70). However, the robust stress response system in the bacteria challenges such efforts and requires a detailed understanding of adaptive strategies (46, 71).

### Terminal oxidoreductases and virulence

The electrons traveling through the ETS ultimately reduce various high-reduction potential molecules like oxygen and nitrate by cognate oxidoreductase enzymes. The diversity of these terminal oxidoreductases enables the utilization of various terminal electron acceptors in the ambient environment (27). The choice of enzyme is often based on the reduction potential governed by bioenergetic hierarchy among the terminal oxidants. For facultative anaerobes like *Escherichia coli*, oxygen is the preferred acceptor, followed by nitrate, nitrite, DMSO, and fumarate (24, 26). The choice of terminal oxidoreductase can impact respiratory efficiency, PMF generation, cellular resource allocation, and redox homeostasis (37, 44, 72). Therefore, this decision has a systems-level metabolic implication and influences pathophysiology.

Given the energetic superiority of oxygen-driven ETS, dioxygen reductases (O_2_REDs) become critical for bacterial survival. Bacteria have evolved several O_2_REDs with varying oxygen affinities and PMF generation abilities to adapt to a range of oxygen concentrations (73). The O_2_RED consisting of heme b and heme d, cytochrome bd oxidase, has a high oxygen affinity and is reported to provide the ability to maintain aerobic respiration in hypoxic environments (73). On the other hand, heme-copper O_2_REDs such as cytochrome-bo_3_ and -aa_3_ oxidase have lower oxygen affinity and are reported to function in oxygen-rich environments. Since different tissue niches have varying oxygen concentrations, such oxidase diversity can be instrumental in bacterial survival. *S. aureus* contains two oxygen-dependent terminal oxidases, cytochrome-bd and -aa_3_ oxidase, which are crucial for systemic infection (74). Deleting the gene *cydB*, which encodes subunit II of cytochrome-bd oxidase, reduces invasion of the heart tissue. On the contrary, in the liver *cydB* deletion does not have an impact, while *qoxB* (encoding subunit I of cytochrome-aa_3_ oxidase) knockout fails to colonize this niche (74). It is plausible that the differences in oxygen tension of these niches promote the utilization of a specific oxidase over another. Interestingly, the O_2_RED expression does not always correlate with the oxygen tension of the niche. In this context, it is essential to note that O_2_REDs usage also depends on ambient oxidative stress and the additional demand for the PMF for transport and motility purposes (75). Such a disconnect between biochemical properties and physiological functions requires detailed investigations.

Cytochrome bd oxidases are also implicated in respiration under nitrosative stress (75). This oxidase has a lower dissociation constant for NO and is less sensitive to NO-mediated inhibition than cytochrome bo_3_ oxidase (76). These properties make bd oxidases the preferred enzyme for several pathogenic species. During infection, *S. enterica* triggers an inflammatory response to gain a competitive advantage over commensal microbes. This inflammatory response can limit the resident microbiota, while *S. enterica* uses NO-resistant bd-oxidase for its luminal expansion. As expected, the bacterial load of *S. enterica* cytochrome bd oxidase deficient strain is reduced in mice colon (77). These mutants also exhibit a lowered respiration rate in the presence of NO stress. The pathological implication of such deficiency can be observed in the improved survival of mice infected with these mutants (78).

The ETS is often extended due to the intermediate cytochrome bc_1_ complex, which can pair with various terminal O_2_REDs to diversify the routes of electron flow. *P. aeruginosa* presents a case of unusually high flexibility in oxygen-dependent terminal oxidases. This opportunistic pathogen contains two major classes of O_2_REDs based on the source of electrons. Cytochrome bo_3_-and cyanide-insensitive quinol oxidase (CIO) accept electrons directly from reduced lipoquinones, whereas cytochrome cbb_3_-1, cytochrome cbb_3_-2, and cytochrome aa_3_-oxidases receive electrons from cytochrome c (79). Interestingly, despite belonging to the cytochrome bd family, CIO has lower oxygen affinity (79). Cytochrome cbb_3_-1 and cytochrome cbb_3_-2 are encoded by two closely placed operons with genes for the four subunits. There are also two partial cbb_3_ operons containing only the catalytic subunits, termed ‘orphan’ cbb_3_ subunits (80). While these orphan oxidases lack the transmembrane monoheme and diheme cytochrome c subunits, the lack of these orphan oxidase subunits results in respiration defects and impacts biofilm development. These mutants are also compromised in reducing the extracellular electron carrier phenazine, suggesting the involvement of these O_2_REDs in cellular and extracellular respiration (81). The high flexibility of terminal oxidases in *P. aeruginosa* might be central to their biofilm lifestyle. As the oxygen tension ranges along the layers of a stratified biofilm, the differential physical and chemical properties of these enzymes may become instrumental (82). This beneficial role in biofilm may be responsible for the strong association of oxidase diversity with *P. aeruginosa* pathogenicity (80, 83).

**Figure 5:**
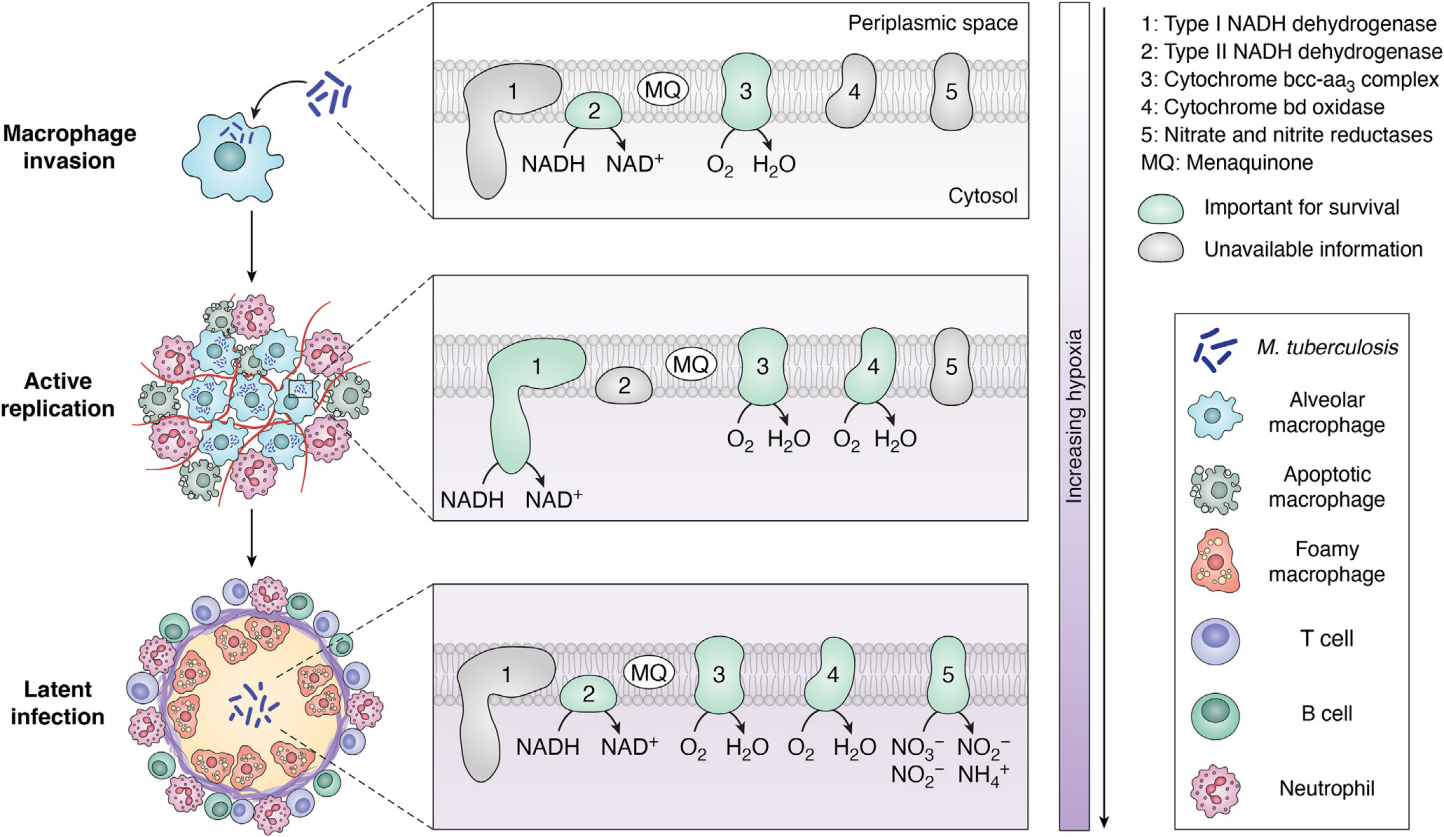
**The respiratory chain plasticity of *Mycobacterium tuberculosis* allows survival in a dynamic niche**. The dehydrogenases and terminal oxidases important for different stages of *M. tuberculosis* infection are shown. As the bacilli invade macrophages, successful invasion requires the activity of type II NADH dehydrogenase and cytochrome bcc-aa_3_ complex (53, 125). At this stage, the ambient oxygen concentration is higher. As infection proceeds, the innate immune response creates a hypoxic environment wherein active replication of *M. tuberculosis* requires the activity of type I NADH dehydrogenase, cytochrome bcc-aa_3_ complex, and cytochrome bd oxidase (126). Finally, during the latent phase of infection, inflammatory byproducts can support anaerobic respiration while trace oxygen is used by high-affinity oxidases. Here, along with the type II NADH dehydrogenase, cytochrome-bcc-aa_3_- and -bd oxidase, nitrate, and nitrite reductases of the bacteria are required to exploit the electron acceptors in this niche (86, 127).

An elegant example of niche exploitation comes from Mtb, where the pathogen thrives in an otherwise hostile environment created by the host immune response (84). Inside activated macrophages infected by Mtb, the reactive oxygen radicals convert NO to nitrate (85). The NarGHI terminal reductase of Mtb can use this nitrate to facilitate ETS. The *narG* deletion mutants show defective propagation inside macrophages. More importantly, wild-type bacilli attain a higher bacterial titer in macrophage cultures when inflammatory conditions are present (85). This suggests that inflammation is not only tolerated but also preferred for mycobacterial proliferation. The NarGHI enzyme further oxidizes nitrate to nitrite. This reaction can also occur independent of the enzyme in inflamed niches by the action of reactive radicals on nitrate. The intelligent pathogenic adaptive approach is evident in Mtb, where, during the latent phase of infection, the bacteria use this nitrite through the NirBD terminal reductase (86). Under hypoxia and with nitrite as a sole nitrogen source in an *in vitro* model of latency, Mtb *nirBD* deletion strains do not survive after the initial replicative phase. *In vivo*, the mutant bacteria cause lower formation of multinucleated giant cells, which are a marker for latency (86). Mtb cells may switch between various terminal oxidases depending on the phase of infection (Fig. 5). Hence, this allows for efficient utilization of the dynamic host environment.

A similar niche adaptation is observed in *Salmonella* spp., which actively migrates toward the site of inflammation (87). For *S. enterica*, energy taxis towards inflammation zones rich in either tetrathionate, nitrate, or aspartate are crucial to its infection cycle (88). It is especially beneficial for gut pathogens to scour alternative electron acceptors, enabling them to outcompete the resident microbiota. While ROS produced due to the inflammatory response is lethal to resident microbiota, *S. enterica* exploits oxidized metabolites like nitrate and tetrathionate, by-products of inflammation, as terminal electron acceptors to maintain oxidative phosphorylation (87). At the same time, the abundance of amino acids, such as aspartate, increases in the microenvironment due to the lysis of the commensal population (89). *S. enterica* uses an aspartate ammonia lyase to break down aspartate to fumarate. Fumarate reductase can further allow the utilization of fumarate as a terminal electron acceptor (89).

The ability to deploy a diverse set of oxidoreductases expands the bandwidth of bacterial survival. Therefore, these respiratory enzymes are being explored as potential drug targets (90, 91). Several candidate molecules inhibiting O_2_REDs, such as imidazopyridine amide (Q203) and 2-aryl-quinolone (CK-2-63), are at various stages of the drug development pipeline (92, 93). Simultaneous suppression of cytochrome bcc-aa_3_ and cytochrome bd oxidases is observed to be a more effective antimycobacterial approach (94, 95). However, cytochrome bcc-aa_3_ inhibiting imidazopyridine amide analog showed a differential activity against Mtb subpopulations residing in and out of granulomas leading to exacerbation of disease (46). Therefore, despite the critical requirement, targeting of oxidoreductase requires an elaborate understanding of disease pathology.

## Concluding remarks

Beyond dehydrogenases and oxidases, the operation of ETS also depends on various mobile electron carriers and complex organization of respiratory enzymes which further enhance respiratory plasticity. Bacterial ETS has diverse free diffusing lipoquinones, differing in their reduction potential, facilitating electron shuttling between dehydrogenases and oxidases (96, 97, 98). These redox-active mobile molecules are also critical in sensing the environment and enabling niche-specific respiratory optimization (96, 99, 100). There are several good reviews detailing the structural and functional diversity of lipoquinones (101, 102). The two primary lipoquinones, ubiquinone and menaquinone, have been reported to influence bacterial pathogenicity. The high reduction potential of ubiquinone makes them more efficient electron carriers in ROS-prone environments and, therefore, their biosynthesis is essential for the survival of *S. enterica* inside infected macrophages (103). Paradoxically, Gram-positive bacteria like *Listeria monocytogenes* that do not have ubiquinone, rely on low-reduction potential quinone, menaquinone, for their survival inside macrophages (104, 105). The ETS components further enhance the scope of metabolic adjustments by physically coming together to form supercomplexes (106, 107, 108). While we are not detailing this concept, the physiological implications of these high-order molecular assemblies are noteworthy in the context of bacterial pathogenesis (109, 110).

The knowledge of respiratory components affecting pathological outcomes is crucial; however, the translational application of this information requires addressing the ‘why’ part of the observations. The mechanistic understanding of the differential usage of various respiratory enzymes is largely superficial in the context of disease progression. For example, the defective survival of Ndh-deficient Mtb inside macrophages is unintuitive given they harbor an alternate type II NADH dehydrogenase, NdhA (53). Interestingly, *S. aureus* solely relying on two type-II NADH dehydrogenases requires both of them for successful infection (111). While the essentiality of both seemingly redundant NADH dehydrogenases is not clear, a recent report suggests that one of these dehydrogenases may utilize NADPH along with NADH (111). In such cases, the subtle differences in enzymatic properties like substrate affinity, optimal pH, temperature, *etc.* may play a deterministic role in their usage under given conditions. Similarly, the availability of terminal electron acceptors may not be the sole factor determining the choice of terminal oxidoreductase. Terminal oxidases like the cytochrome-bd oxidase also have ROS-mitigation abilities critical for withstanding host immune assaults (75).

In this review, we have restricted ourselves to ETS plasticity; however, other components of bioenergetics, like glycolytic and fermentative pathways, also significantly contribute to energy homeostasis. There are several extensive reviews on the pathogenic implications of these pathways (8, 112, 113). As outlined in these reviews, bioenergetics is central to bacterial pathogenesis and survival. However, the bulk of information in the field comes from a select few bacterial species. The broadening of the range of bacterial species can provide pathophysiological implications for the understudied metabolic diversity.

While it is gratifying to note many outstanding studies in recent times, there is a need for greater effort in the context of the looming threat of an antimicrobial resistance crisis. Towards this, energy metabolism is being explored as a target space for newer antimicrobial development (69, 114). However, the remarkable tenacity in the respiratory system necessitates an in-depth familiarity with the bioenergetic adaptability for advancing translation in this area. While laboratory-based adaptive evolution experiments reveal compensatory mechanisms, the knowledge disconnect between proximal and distal responses within complex host environments requires careful examination (71, 115, 116). The details emerging from these studies can facilitate efficient and informed targeting of bacterial energetics. Additionally, the cohesive understanding of the bioenergetic system in relation to microenvironments can facilitate the development of antibiotic-independent treatment approaches, especially engineered biotherapeutics. The use of probiotics is being proposed as an attractive approach in reducing dependency on antibiotics (117). An exciting development would be the exploitation of the knowledge of host-pathogen metabolic interactions as a design principle for developing synthetic probiotics. Therefore, there is a need for more integrative studies detailing the mechanistic nexus of niche, nutrient, and metabolic networks.

## Data availability

All supporting data are provided within the manuscript, supplementary data and supplementary tables.

## Conflict of interest

The authors declare that they have no conflicts of interest with the contents of this article.
